# Biochemical basis of permethrin resistance in *Anopheles arabiensis* from Lower Moshi, north-eastern Tanzania

**DOI:** 10.1186/1475-2875-9-193

**Published:** 2010-07-07

**Authors:** Johnson Matowo, Manisha A Kulkarni, Franklin W Mosha, Richard M Oxborough, Jovin A Kitau, Filemoni Tenu, Mark Rowland

**Affiliations:** 1 Pan-African Malaria Vector Research Consortium www.pamverc.org; 2 KCM College of Tumaini University, P.O.Box 2240, Moshi, Tanzania; 3 Department of Biology, University of Ottawa, 30 Marie Curie, Ottawa, ON, KIN 6N5, Canada; 4 London School of Hygiene and Tropical Medicine, Keppel Street, London WC1E 7HT, UK; 5 National Institute for Medical Research, Amani Medical Research Centre, P.O. Box 81, Muheza, Tanzania

## Abstract

**Background:**

Development of resistance to different classes of insecticides is a potential threat to malaria control. With the increasing coverage of long-lasting insecticide-treated nets in Tanzania, the continued monitoring of resistance in vector populations is crucial. It may facilitate the development of novel strategies to prevent or minimize the spread of resistance. In this study, metabolic-based mechanisms conferring permethrin (pyrethroid) resistance were investigated in *Anopheles arabiensis *of Lower Moshi, Kilimanjaro region of north-eastern Tanzania.

**Methods:**

WHO susceptibility test kits were used to detect resistance to permethrin in *An. arabiensis*. The levels and mechanisms of permethrin resistance were determined using CDC bottle bioassays and microplate (biochemical) assays. In bottle bioassays, piperonyl butoxide (PBO) and s,s,s-tributyl phosphorotrithioate (DEF) were used as synergists to inhibit mixed function oxidases and non-specific esterases respectively. Biochemical assays were carried out in individual mosquitoes to detect any increase in the activity of enzymes typically involved in insecticide metabolism (mixed function oxidases, α- and β-esterases).

**Results:**

*Anopheles arabiensis *from the study area was found to be partially resistant to permethrin, giving only 87% mortality in WHO test kits. Resistance ratios at KT_50 _and KT_95 _were 4.0 and 4.3 respectively. The permethrin resistance was partially synergized by DEF and by PBO when these were mixed with permethrin in bottle bioassays and was fully synergized when DEF and PBO were used together. The levels of oxidase and β-esterase activity were significantly higher in *An. arabiensis *from Lower Moshi than in the laboratory susceptible strain. There was no difference in α-esterase activity between the two strains.

**Conclusion:**

Elevated levels of mixed function oxidases and β-esterases play a role in detoxification of permethrin in the resistant *An. arabiensis *population of Lower Moshi.

## Background

Resistance to pyrethroids and other insecticides is an important threat to the control of malaria in Africa [1-3]. Early detection of insecticide resistance enables more rational selection of insecticides. In recent years, Anopheline mosquitoes in many parts of Africa have become resistant to pyrethroids, partly in response to agricultural application or run off of insecticides into mosquito breeding sites [2,4-6], but increasingly in response to selection pressure resulting from the scaling up of long-lasting insecticide-treated nets and indoor residual spraying as malaria prevention tools [3,7-11]. Development of resistance may necessitate switching to an alternative class of insecticide to enable resumption of control [3]. Early detection of resistance facilitates more rational selection of insecticides or may enable timely introduction of resistance management strategies [12].

There are two broad mechanisms by which insect pests develop resistance to insecticides. They may produce increased quantities of enzymes, which either metabolize the insecticide or sequestrate the molecules so they cannot function. The second mechanism involves mutation of the insecticide target-site. This effectively blocks the action of the insecticide. Both types of mechanism have been studied in various species of insects [12-16].

Levels of insecticide resistance in field populations of vectors have been shown to vary over relatively small geographical areas and over different seasons [5,17]. In Africa, the major foci of pyrethroid resistance are found in the western and central regions, especially in cotton growing areas where pyrethroids have been applied intensively against cotton pests [2]. In *Anopheles gambiae *populations of these areas, reduced target site sensitivity arising from a single point mutation in the sodium channel gene (*kdr*) has been implicated as the predominant mode of resistance [16], although increased levels of detoxifying enzymes may also play a role [4]. An increase in permethrin tolerance in *An. gambiae *in Kenya due to localized use of ITNs has been associated with both target site insensitivity and elevated levels of detoxifying mixed function oxidases (MFOs) [18]. In South Africa, *Anopheles funestus *developed pyrethroid resistance after recurrent campaigns of indoor residual spraying with deltamethrin [3]. Detection of the West African *kdr *mutation at low frequency in *An. arabiensis *in Lower Moshi, north-eastern Tanzania [19] and the West African *kdr *mutation in *An. gambiae *in Uganda [20] suggests the *kdr *gene has spread across the African continent.

The standard method for detecting resistance in populations of mosquito vectors is the WHO susceptibility test [21]. Application of a discriminating concentration that distinguishes susceptible from resistant mosquitoes allows accurate detection of resistance when the gene is dominant. But when resistance is recessive or present at low frequency discriminating-dose tests based on mortality may lack the necessary precision. In such situations, the use of knockdown time - expressed as the time taken for 50% of individuals to be knocked down - may prove a more sensitive indicator of resistance [22]. Additional information on resistance mechanisms can be derived from use of synergists, chemicals that inhibit the enzymes responsible for insecticide metabolism [9,12-14,23]. By combining synergist with insecticide in bioassay tests the resistant mosquitoes will return to apparent susceptibility if the inhibited enzyme is responsible for resistance [23]. Biochemical assays have been developed that measure enhanced levels of detoxification enzymes responsible for resistance. These may be performed on individual mosquitoes allowing more sensitive detection of resistance [24-26]. The aim of this study was to investigate the biochemical mechanisms of permethrin resistance in field populations of *An. arabiensis *from Lower Moshi in Kilimanjaro region of north-eastern Tanzania. In this irrigated rice growing area, *An. arabiensis *is the predominant vector of malaria [27]. Preliminary studies suggest an absence of *kdr*-based mechanisms [19] while an oxidase-based mechanism may be present [28]. Bioassay and biochemical assays were used to determine the contributions of both mixed function oxidases and non-specific esterases to the observed permethrin resistance.

## Methods

### Study area and mosquito collection sites

The study was carried out on mosquitoes from Lower Moshi, an intensive rice-irrigation area, 37°20'E 3°21'S and 700 meters altitude, south of Mount Kilimanjaro in north-eastern Tanzania. Most of the population in the area is engaged in agriculture. Two rivers, the Njoro and the Rau provide water for irrigation. There are two growing seasons, the main one from June to October and the second one involving sporadic cultivation of rice from September to February. *Anopheles arabiensis *adults were collected during January-April 2007 from the villages of Mabogini, Rau-Kati, and Chekereni.

### Mosquito strains

The two strains used in this study were *An. arabiensis *Dondotha, a laboratory insecticide susceptible strain, and *An. arabiensis *wild, a field strain from Lower Moshi, resistant to permethrin, established from collections of indoor resting adult mosquitoes from animal houses. The mosquitoes were transferred to the insectary and females induced to lay eggs. The F1 progeny was divided into two sub samples: one sub sample was stored at -80°C for biochemical analysis while the other was used for insecticide and synergist bioassays.

### Diagnostic resistance tests

Diagnostic tests were conducted on adult *An. arabiensis *using WHO susceptibility test kits [29]. Batches of 25 blood-fed mosquitoes taken directly from field collections were exposed to 0.75% permethrin test papers according to WHO procedures with the cylinder placed in a vertical position [21]. Knockdown was recorded every ten minutes during the 1 hour exposure. At the end of the exposure period, mosquitoes were transferred to recovery tubes and provided with glucose solution, held for 24 hours after which mortality was recorded. Four test batches (100 mosquitoes) and one control (25 mosquitoes) were tested during each test run which was replicated ten times; hence 1000 wild females were tested for permethrin resistance status. Tests were carried out in parallel on blood-fed insecticide-susceptible adults of the Dondotha stain of *An. arabiensis*

### Synergy tests

Bottle bioassay calibration was conducted to determine appropriate concentrations of insecticide and synergist. Glass bottles of 250 ml capacity were coated with three concentrations of permethrin (12.5, 25 or 50 μg per bottle) according to the method of Brogdon and McAllister [23] and tested against the susceptible strain for 1-hour to determine the baseline response to permethrin. Ten replicates of ten mosquitoes were tested against each concentration for one hour during which knockdown was recorded at 5 min intervals. The ideal test concentration of insecticide is the lowest one that gives a time-mortality response from 0 to 100% mortality over a convenient test period [23]. To verify that the concentrations of synergist were below toxic levels, a series of concentrations of s,s,s-tributyl phosphorotrithioate DEF (range 62.5-250 μg per bottle) and piperonyl butoxide PBO (range 100-400 μg per bottle) were tested against the susceptible strain for 60 min.

Synergy tests were conducted on adult progeny of wild *An. arabiensis *from Lower Moshi and on the laboratory susceptible strain. The glass bottles were coated with permethrin with or without a synergist as described by Brogdon *et al *[23]. PBO, an inhibitor of mixed function oxidases, and DEF, an inhibitor of non-specific esterases, were the synergists used. Four treatments were compared during each test run: permethrin alone, permethrin plus DEF, permethrin plus PBO or permethrin plus PBO and DEF together (plus positive and negative controls). Ten non-blood-fed females, three- to five-day old were used in each replicate, and each treatment was tested ten times. Knockdown was recorded at 5-minute intervals for 60 minutes. Mosquitoes were then transferred to holding cups, supplied with glucose solution and mortality recorded after 24 hours.

### Biochemical assays

Biochemical assays were used to quantify the levels of mixed function oxidase and non-specific esterase activity in individual mosquitoes. Individual 2-5 day old *An. arabiensis *adults, reared under insecticide-free conditions and stored at -80°C, were homogenized manually in sodium phosphate buffer (pH 7.2) inside a 1.5 ml eppendorf tubes. For the oxidase assays individuals were homogenized in 100 μl of 0.0625M sodium phosphate buffer (pH 7.2), which was then diluted by adding 1,400 μl of the sodium phosphate buffer. For esterase assays, individuals were homogenized in 20 μl of 0.02M sodium phosphate buffer (pH 7.2) to which 100 μl of distilled water was added. A hundred microlitres of sodium phosphate buffer pH 7.2 was added to the aliquots of mosquito homogenates and 200 μg of 3, 3, 5', 5'-tetramethhyl benzidine (TMBZ) solution (0.01 g of 3, 3, 5', 5'-tetramethhyl benzidine in 5 ml of absolute methanol, mixed with 0.25M sodium acetate buffer pH 5.0) was added. Twenty-five microlitres of 3% hydrogen peroxide was added and the mixture was left for two hours at room temperature. The oxidase enzyme activity was then read at 630 nm. For the esterase assays 20 μl of α-naphthyl acetate solution (1 ml of 30 mM α-naphthyl acetate in acetone in 99 ml of 0.02M phosphate buffer pH 7.2) and 200 μg β-naphthyl acetate solution (prepared as for α-naphthyl acetate solution) were added to replicates of mosquito homogenates. The enzyme reaction was run for two minutes at room temperature before the addition of 50 μl of Fast blue stain solution. The absorbance value for each well was determined at 570 nm.

### Statistical analysis

Mortality in WHO resistance tests was corrected for control mortality using Abbott's formula [30]. KT_50 _and KT_95 _values in the bottle bioassays and resistance ratios between wild and susceptible strains were estimated using probit analysis (Polo Plus 1.0, LeOra Software). Enzyme expression levels between strains were compared using t-tests.

## Results

### WHO resistance tests

**Figure 1:**
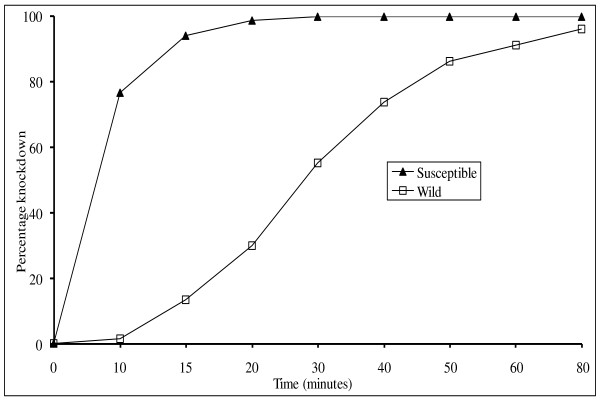
**Percentage knockdown over time for F1 generation of wild mosquitoes and susceptible strain**. Ten replicates were run for each strain (n = 1069 for F1 *Anopheles arabiensis *(susceptible), n = 1011 for F1 *Anopheles arabiensis *(wild).

Percentage mortality to permethrin in susceptibility tests conducted on wild females from the villages of Mabogini, Rau Kati, and Chekereni ranged from 75.5% to 96.1% and showed an average of 87% mortality (corrected for control). Mortality of the laboratory susceptible strain was 100%. Figure 1 shows mean percentage knockdown at different time intervals.

Knockdown took considerably longer in tests on the wild strain: the time for 50% to be knocked down took 28 min in the wild strain but took less than 7 min in the susceptible strain. The resistance ratios at KT_50 _and KT_95 _were 4.0 and 4.3 respectively (Table 1).

**Table 1:** Susceptibility tests with permethrin

	Knockdown time (minutes) for permethrin		%24 h mortality [Corrected for control]	
		
	**KT**_**50 **_**(95% C.I)**	**KT**_**95 **_**(95% C.I)**	Permethrin	DDT
Susceptible	7 (5 - 8)	16 (15 - 18)	100 [100]	100 [100]
Wild	28 (27 - 29)	69 (65 - 74)	88 [87]	100 [100]

Resistance ratios	4.0	4.3		

### Bottle bioassays and synergy tests

The best-fit straight line (R^2 ^= 0.91) was obtained with 12.5 μg/bottle permethrin. The highest concentration of synergist that produced no lethal effect was 200 μg PBO per bottle and 125 μg DEF per bottle. These concentrations of insecticide and synergist were used in subsequent synergy tests. Following exposure to permethrin for 60 minutes, the % mortality rate of 3-5 day old female mosquitoes of *An. arabiensis *wild was consistently lower than that observed for the susceptible strain (Table 2). Knockdown time at KT_50 _and KT_95 _was higher in the wild than in the susceptible strain and the resistance ratios were 2.7 folds lower in the bottle bioassay than in the WHO resistance tests.

**Table 2:** Bottle bioassay tests with permethrin

	Knockdown time (minutes)		% mortality
		
	**KT**_**50 **_**(95% C.I)**	**KT**_**95 **_**(95% C.I)**	
Susceptible	17 (15 - 19)	30 (27 - 35)	95
Wild	26 (24 - 27)	49 (45 - 54)	78

Resistance ratios	1.5	1.6	

The p-value for KT_50 _and KT_95 _< 0.001

**Figure 2:**
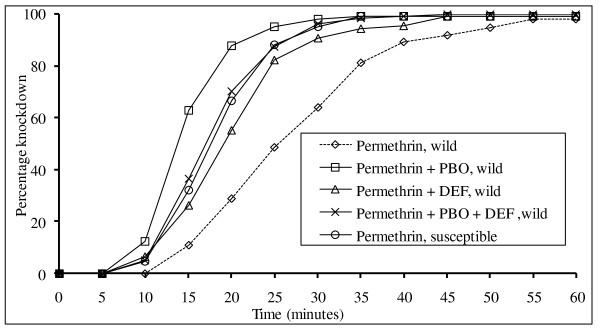
**Mortality over time for F1 generation wild mosquitoes exposed to permethrin alone, permethrin + PBO, permethrin + DEF, permethrin + PBO + DEF (plus permethrin test of susceptible mosquito strain)**. Each curve is represented by data from ten replicates (n~100 mosquitoes).

When F1 *An. arabiensis *mosquitoes were exposed to permethrin plus PBO or DEF the % knockdown was much higher than when exposed to permethrin alone (Figure 2). Permethrin plus PBO consistently recorded higher % knockdown than permethrin plus PBO and DEF in replicates. The synergy ratio was relatively higher when permethrin was synergized by PBO than when it was synergized by either DEF or both PBO and DEF (Table 3).

**Table 3:** Synergy tests with wild mosquitoes

Treatment	Knockdown time (minutes)		Synergy ratios	
		
	**(KT**_**50**_**)**	**(KT**_**95**_**)**	**(KT**_**50**_**)**	**(KT**_**95**_**)**
Permethrin alone	26 (24-27)	49 (45-54)		
Permethrin + PBO	15 (13-16)	23 (21-25)	1.7	2.1
Permethrin + DEF	20 (18-22)	33 (30-37)	1.3	1.5

Permethrin + PBO + DEF	18 (16-20)	28 (26-32)	1.4	1.8

The p-value for KT_50 _and KT_95 _< 0.001

### Microplate assays of esterase and oxidase activity

**Figure 3:**
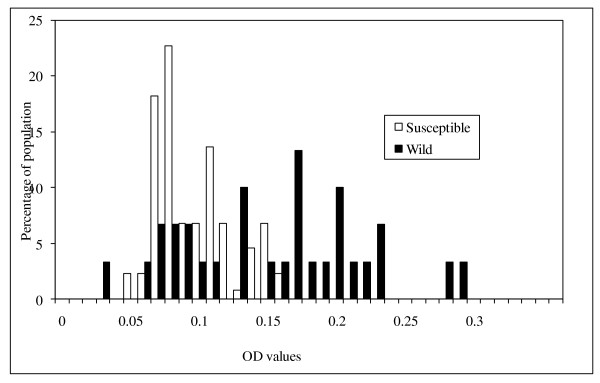
**The distribution of activity of mixed function oxidases in wild and susceptible *Anopheles arabiensis *adult females**. Absorbance values recorded at 630 nm.

**Figure 4:**
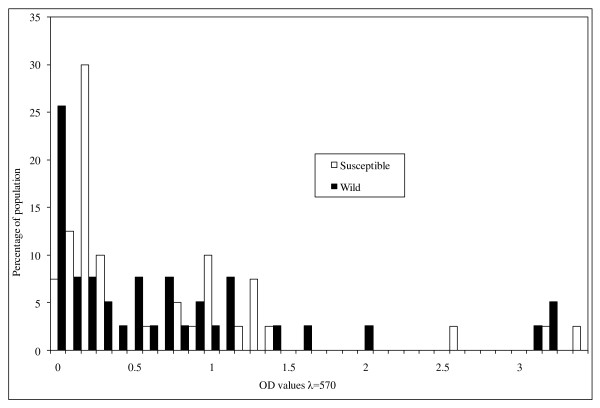
**The distribution of activity of α-esterases in wild and susceptible *Anopheles arabiensis *adult females**. Absorbance values recorded at 570 nm.

**Figure 5:**
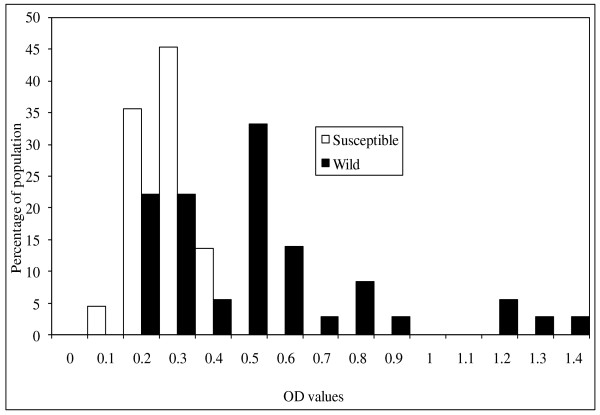
**The distribution of activity of β-esterases in wild and susceptible *Anopheles arabiensis *adult females**. Absorbance values recorded at 570 nm.

The means and confidence intervals for the optical density values for α-esterases, β-esterases and mixed function oxidases are shown in table 4. The mean levels of mixed function oxidase and beta β-esterase activity were significantly higher for *An. arabiensis *wild than for the susceptible strain. However, the mean levels of α-esterase activity were not significantly different between wild and susceptible strains. Figures 3, 4 and 5 show the distribution of MFO, α-esterase and β-esterase activity respectively. The OD cut off points that discriminate between susceptible and wild strains were 0.165 for MFOs and 0.45 for β-esterases; the proportion of wild individuals scoring above these thresholds were 50% (15/30) for MFOs and 44% (17/39) for β-esterases. These biochemical proportions are higher than the proportion of mosquitoes that showed phenotypic resistance in WHO and bottle bioassay resistance tests which suggests that a combination of elevated MFO and elevated esterase activity might be required to give rise to phenotypic resistance.

**Table 4:** Microtitre plate assays of enzyme activity

	Optical Density values: Mean (CI)		
	
	α - esterase (n = 39)	β - esterase (n = 22)	Oxidase (n = 30)
Susceptible	0.79 (0.52-1.05)	0.26 (0.23-0.30)	0.12 (0.11-0.13)
Wild	0.84 (0.58-1.11)	0.48 (0.36-0.60)	0.17 (0.20-0.14)

Difference	-0.05* (-0.45, 0.34)	-0.22** (-0.35, -0.08)	-0.06** (-0.08, -0.03)

## Discussion

The study showed that a low frequency of permethrin resistance mediated by MFOs and β-esterases is present in *An. arabiensis*, the predominant malaria vector of Lower Moshi. The permethrin resistance is probably caused by the agricultural use of insecticides, especially in the rice fields, as permethrin-treated nets are not widely used in Lower Moshi for protection against mosquitoes. In Kenya, the localized use of permethrin-impregnated nets in Kisumu did increase the permethrin tolerance of the local population of *An. gambiae sensu stricto *[18].

However, there was no evidence that it reduced the efficacy of permethrin-impregnated nets as a malaria control measure [31]. The present frequency of resistance in Lower Moshi appears to not impair the effectiveness of permethrin treated nets. A recent field trial in Moshi shows that while such treated nets kill relatively few host-seeking *An. arabiensis *that enter local houses the nets continue to provide personal protection through the strong excito-repellent activity of permethrin [32]. Permethrin-treated nets in the form of the Olyset LLIN are now being scaled up in Tanzania as national malaria control policy and if the increased coverage selects further for permethrin resistance the effectiveness of the LLIN strategy may ultimately be undermined. Stump *et al *[33] recently reported pyrethroid resistance in the form of *kdr *from an area of long-term ITN use in western Kenya. Applying SSOP-ELISA method, Kulkarni *et al *[19] reported *kdr *(western variant) at low frequency in *An. arabiensis *just a few kilometers from the present study area. Only 2 out of 642 mosquitoes (0.3%) appeared to carry *kdr *mutation and only in heterozygous genotype. The *kdr *mechanism results from mutations in the voltage-gated sodium channels, the target-site for DDT and pyrethroids. Furthermore, there is no DDT resistance in the study area as recorded in Table 1. The absence of DDT resistance and the presence of permethrin resistance suggest insecticide detoxification by enzymes to be the more important mechanism for permethrin resistance in Lower Moshi. At the moment *kdr *is too rare to be important but further selection of combined metabolic and site insensitivity resistance by wider use of LLINs might constitute a grave threat.

Mixed function oxidases (MFOs) and non-specific esterases (NSEs) are commonly involved in the detoxification of permethrin [34-36]. Elevated levels of such enzymes are known to enhance permethrin resistance [24]. Exposure of *An. arabiensis *(wild) to permethrin plus DEF or PBO synergists inhibited non-specific esterase and oxidase activities and led to higher mortality rates than exposure to permethrin alone. This indicates involvement of both oxidases and esterases in conferring permethrin resistance. Exposure to permethrin with PBO and DEF together resulted in an exposure time/mortality curve equivalent to that of the susceptible strain of *An. arabiensis *exposed to permethrin alone, with KT_50 _value that is within the KT_50 _95%CI of the susceptible strain. This implies total inhibition of permethrin resistance in the wild strain.

Higher mortality produced in *An. arabiensis *exposed to permethrin plus PBO relative to the susceptible strain exposed to permethrin could be due to significant increase in the cuticular penetration rate of permethrin. It suggests that the synergist might have caused synergism by an acceleration of permethrin across the cuticle on addition to increased permethrin detoxification in the resistant wild mosquito strain. According to Sun and Johnson [37], some synergists cause synergism solely by an acceleration of insecticide across the cuticle (quasi-synergism). The study by Kennaugh *et al *[38] shows no evidence for increased permethrin detoxification in the resistant strain of *Helicoverpa armigera *(cotton bollworm) although permethrin resistance could be eliminated with PBO. This suggests accelerated permethrin penetration through the cuticle as a possible mechanism for that synergism. In 1995, Gunning *et al *[39] reported an increased rate of esfenvalerate penetration in the presence of PBO in *Helicoverpa armigera*. The lower mortality observed when *An. arabiensis *was exposed to permethrin with PBO and DEF together compared to when exposed to permethrin plus PBO is probably due to antagonistic effect of DEF to PBO on cuticular penetration of the permethrin. In this case, DEF might have reduced penetration of the permethrin, hence reducing the time for its detoxification. It could also be due to esterase inhibition by PBO resulting in the loss of esterase-mediated sequestration of the permethrin. The new assay devised by Khot *et al *[40] reveals the blockade of esterases by PBO. This could not be the case in this mosquito population since the observed mortality when permethrin was mixed with PBO alone is significantly different from the observed mortality when permethin was mixed with both PBO and DEF.

Elevation of one or more broad substrate spectrum esterase is a common mechanism of insecticide resistance in *Culex *species [41]. Although less common in Anopheles, elevated esterases have been documented in pyrethroid-resistant *An. gambiae *from Kenya [14,15]. In the present study, the association of elevated mixed function oxidases and non-specific esterases was confirmed biochemically using oxidase and esterase detection assays. The proportion of *An. arabiensis *wild with enzyme levels higher than that for the susceptible strain did not equate with the phenotypic expression of resistance in WHO test kits or bottle bioassays. This may mean that elevated MFOs and esterases must occur together to give rise to phenotypic resistance. This may also indicate that biochemical tests are unable to correlate fully with phenotypic resistance or serve as a reliable indicator of metabolic resistance.

## Conclusion

β-esterase mediated hydrolysis and oxidative detoxification by monooxygenases are the predominant mechanisms of permethrin resistance in adult *An. arabiensis *of Lower Moshi, north-eastern Tanzania. The low frequency of phenotypic resistance is probably due to the use of insecticides in rice plantations or use of permethrin-treated nets. At the present level of resistance (0.16% allele frequency for L104F *kdr *genotype), permethrin-treated nets remain efficacious, but the further selection of metabolic and *kdr *mechanisms, also present in the study area, may in combination prove a graver threat. There may be scope for effective use of LLINs that incorporate PBO in the fibres. Resistance was associated with elevated esterases and MFOs but chemical assays that detect elevated levels in individual insects did not necessarily correlate with phenotypic resistance as detected by WHO resistance test kits or CDC bottle bioassays.

## Competing interests

The authors declare that they have no competing interests.

## Authors' contributions

Johnson Matowo developed the study design, performed bottle and microplate assays, carried out data analysis and interpretation and wrote the first drafts of the manuscript. Manisha Kulkarni contributed to study design, provided technical help, training and support on biochemical techniques, and revised the manuscript. Mark Rowland obtained funding for the work, interpreted the results and substantially revised the manuscript. Filemoni Tenu, Jovin Kitau and Richard Oxborough participated in data processing, statistical analysis and interpretation. Franklin Mosha co-designed the study and provided critical review of the manuscript. All authors have read and approved the final manuscript.
